# Supplementary material for: Evaluating 10 years of state-funded GP training in GP offices in Switzerland
Source: PLoS One. 2020 Aug 17;15(8):e0237533. doi: 10.1371/journal.pone.0237533 (PMC7430752; doi:10.1371/journal.pone.0237533)
Supplement: S1 Data — (DOCX) [file pone.0237533.s001.docx]

***Was geschah nach der Praxisassistenz?***
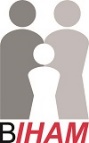


**Supporting information 1.** Original survey used

**Praxistätigkeit Haben Sie die Praxistätigkeit als Hausärztin/Hausarzt in der Schweiz aufgenommen?**

☐ Nein ☐ Ja

t

**Angaben zur Praxistätigkeit**

**Berufswahl**

**Welche Aussage in Bezug auf die Berufswahl Bitte machen Sie ein paar Angaben zu Ihrer Praxistätigkeit trifft am ehesten auf Sie zu?** Beginn der Praxistätigkeit (Jahr)

☐ Ich werde als Hausärztin/-arzt tätig sein, Postleitzahl der Praxis die Praxisaufnahme ist schon geplant

☐ Ich bin immer noch auf dem Track **Ihr Arbeitspensum in Halbtagen pro Woche**  Hausarztmedizin, habe aber noch keine ☐ 1 ☐ 2 ☐ 3 ☐ 4 ☐ 5 ☐ 6 ☐ 7 ☐ 8 ☐ 9 ☐ 10 und mehr feste Planung zur Praxisaufnahme ***🡪 zu A***

☐ Ich bin unsicher, ob die Hausarztmedizin **In welchem Praxismodell arbeiten Sie?**  das Richtige für mich ist und ☐ Einzelpraxis ☐ Gruppenpraxis < 3 ÄrztInnen konnte mich noch nicht entscheiden ***🡪 zu C*** ☐ Gruppenpraxis >= 3 ÄrztInnen

☐ Ich habe mich für eine andere

Tätigkeit als Hausarztmedizin entschieden ***🡪 zu B*** **Haben Sie die Praxis übernommen oder sind Sie dort**

☐ Keine der obigen Aussagen trifft auf mich zu, **eingestiegen, wo Sie die Praxisassistenz absolviert haben?**

sondern ***🡪 zu B*** ☐ ja ☐ nein

**Geplante Praxistätigkeit**

**Bitte machen Sie ein paar Angaben zu Ihrer Praxistätigkeit:**

Voraussichtlicher Beginn der Praxistätigkeit (Jahr) Postleitzahl der Praxis

**Welches Arbeitspensum streben Sie an (Angabe in Halbtagen pro Woche)**

☐ 1 ☐ 2 ☐ 3 ☐ 4 ☐ 5 ☐ 6 ☐ 7 ☐ 8 ☐ 9 ☐ 10 und mehr

**Planen Sie die Praxis zu übernehmen oder dort einzusteigen,**

**wo Sie eine Praxisassistenz absolviert haben?**

☐ ja ☐ nein

**Praxisassistenz Wie wichtig war für Sie die *Praxisassistenz* in der Entscheidung Hausärztin/-arzt zu werden?** ☐ sehr unwichtig ☐ unwichtig ☐ neutral ☐ wichtig ☐ sehr wichtig

**Wie wichtig war für Sie der *Einfluss Ihres/Ihrer damaligen Lehrpraktikers/-praktikerin* in der Entscheidung Hausärztin/-arzt zu werden?**

☐ sehr unwichtig ☐ unwichtig ☐ neutral ☐ wichtig ☐ sehr wichtig

**A. Gründe für Hausarztmedizin**

**Was waren Ihre drei wichtigsten Gründe Hausärztin bzw. Hausarzt zu werden?**

***🡪 zu C***

**B.Gründe gegen die Hausarztmedizin**

**Was waren die drei wichtigsten Gründe nicht Hausärztin bzw. Hausarzt zu werden?**

**Andere Tätigkeit Für welche Tätigkeit haben Sie sich entschieden?**

**Was trifft für Sie zu?** ☐ Ich halte es für nicht ausgeschlossen, dass ich später noch in die Hausarztmedizin wechsle ☐ Ich denke nicht, dass ich später in die Hausarztmedizin wechseln werde ☐ anderes / Kommentar

**C. Abschluss**

**Haben Sie noch Anmerkungen oder Kommentare?**

**Geben Sie erneut Ihre Emailadresse ein, wenn Sie über die Umfrageresultate informiert werden möchten.**

***What happend after GP training?***
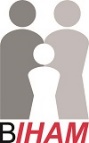


**Supporting information 1.** Translation of the original survey used

**Staring as GP
Have you started working as GP in Switzerland?**

☐ No ☐ Yes

**Characteristics of your GP practice**

**Career choice**

**Which Answer below describes your current Please specify your GP activity career choice best?** Start of working as GP (year)

☐ I will start working as GP ZIP code of your working place and the time point is already defined

☐ I am still on track to become GP **How many half-days per week do you work?**

but the time point is not yet defined ☐ 1 ☐ 2 ☐ 3 ☐ 4 ☐ 5 ☐ 6 ☐ 7 ☐ 8 ☐ 9 ☐ 10 or more

***🡪 Move to A***

☐ I am unsure if GP is **Define your practice model**  the right choice for me ☐ solo ☐ group < 3 GPs and I am undecided ***🡪 Move to C*** ☐ group >= 3 GPs

☐ I decided for a career

outside general practice ***🡪 Move to B*** **Did you start working as GP at the same practice**

☐ none of the above options **where you completed your GP training program?**

but ***🡪 Move to B*** ☐ Yes ☐ No

**Planned GP activity**

**Describe your planned activity:**

Planed time point to start (year)

ZIP code of your planned working place

**How many half-days per week do you plan to work?**

☐ 1 ☐ 2 ☐ 3 ☐ 4 ☐ 5 ☐ 6 ☐ 7 ☐ 8 ☐ 9 ☐ 10 or more

**Do you plan to start working as GP at the same practice**

**where you completed your GP training program?**

☐ Yes ☐ No

**GP training
How important was your GP training in GP practice
in your decision to become GP?**☐ very unimportant ☐ unimportant ☐ neutral ☐ important ☐ very important

**How important was the influence of the formar GP trainer
in your decision to become GP?**

☐ very unimportant ☐ unimportant ☐ neutral ☐ important ☐ very important

**A. Reasons to choose General Practice**

**Name your three most important reasons to become GP**

***🡪 Move to C***

**B.Reasons against choosing General Practice**

**Name your three most important reasons not to become GP**

**Other career choice
For which activity did you decide?**

**Choose what describes your situation best**☐ I cannot rule out that I might come back to choose working as GP
☐ I can rule out that I'd come back to choose working as GP
☐ others/comment

**C. End**

**Do you have comments or suggestions?**

**Enter your email adress if you liked to be informed about the study results**
