# Supplementary material for: Evaluating 10 years of state-funded GP training in GP offices in Switzerland
Source: PLoS One. 2020 Aug 17;15(8):e0237533. doi: 10.1371/journal.pone.0237533 (PMC7430752; doi:10.1371/journal.pone.0237533)
Supplement: Attachment — Submitted filename: Responsetoreviewers.docx [file pone.0237533.s003.docx]

Professor Sven Streit, MD, MSc, PhD

Professor in Primary Care

Head of Interprofessional Primary Care
Institute of Primary Health Care Bern (BIHAM)

University of Bern
Mittelstrasse 43
3012 Bern

Switzerland
Tel +41 31 631 58 75
Email: sven.streit@biham.unibe.ch

**Revision of manuscript ID:** PONE-D-20-09608

Dear Dr Soundy, dear PLOS ONE Editorial Board,

We were invited to submit a revised version of our manuscript:

**“Evaluating 10 years of state-funded GP training in GP offices in Switzerland**

We would like to thank you for the opportunity to revise and resubmit a revised version of our manuscript.

We believe the comments and suggestions made by both reviewers helped to improve our manuscript and we were happy to implement them in our manuscript. Please find a detailed response to the questions and comments raised by reviewers in our point-by-point response below.

Again, we thank you for the opportunity to strengthen our manuscript with your valuable comments. We hope that the revisions persuade you to accept our submission.

Sincerely,

Sven Streit

**Journal Requirements:**

**When submitting your revision, we need you to address these additional requirements.**

**1. Please ensure that your manuscript meets PLOS ONE's style requirements, including those for file naming. The PLOS ONE style templates can be found at https://journals.plos.org/plosone/s/file?id=wjVg/PLOSOne_formatting_sample_main_body.pdf and https://journals.plos.org/plosone/s/file?id=ba62/PLOSOne_formatting_sample_title_authors_affiliations.pdf**

Response: we adapted our revision accordingly.

**2. Please include additional information regarding the survey or questionnaire used in the study and ensure that you have provided sufficient details that others could replicate the analyses. For instance, if you developed a questionnaire as part of this study and it is not under a copyright more restrictive than CC-BY, please include a copy, in both the original language and English, as Supporting Information.**

Response: We added our original survey and an English version as supporting information and added on line 112:

" The original survey and an English version is available (Supporting Information 1). "

**3. Please amend either the abstract on the online submission form (via Edit Submission) or the abstract in the manuscript so that they are identical.**

Response: we made sure both the abstract in the online submission and the manuscript are identical.

**4. Your ethics statement must appear in the Methods section of your manuscript. If your ethics statement is written in any section besides the Methods, please move it to the Methods section and delete it from any other section. Please also ensure that your ethics statement is included in your manuscript, as the ethics section of your online submission will not be published alongside your manuscript.**

Response: We moved the ethics statement as requested to the methods section.

**Editor Comments**

**1. Can you detail steps taken in survey development if it was a new survey e.g.,**

**Questionnaire design and pretesting**

Response: We agree that further clarification as rightly pointed out are mandatory. We used ta shortened version of he most established national questionnaire issued by the WHM foundation (our reference 7). We believe for readers this additional information is helpful so we added on page 6:

" Our survey was a shortened version from the questionnaire that is used by the WHM foundation (7). WHM has a long tradition to evaluate GP training program in >800 Swiss residents and is the most widely used questionnaire in Switzerland. The design of the questionnaire is regularly under audit for futher improvements. "

**2. Can you consider non-sampling error**

Response: There is always a risk of a non-sampling error when doing survey. But as pointed out in line 226 of our limitation section: only 8% of invited GP trainees did not answer to the survey why we believe the risk of a non-sampling error is small.

**3. Can you use STROBE to detail sections of the method and identify important aspects like bias, confounding variables etc.**

Response: We provide a STROBE checklist to make sure all aspects mentioned are covered in our manuscript.

**Reviewer 1**

**1. Thank you for your insight in the influences of the GP-training program on (future) GP's. I have some questions for further optimizing your paper:
- I would like to advise you to consider using a translation agency or a native speaker in order to optimize the use of language in your paper.**

Response: Thank you for reviewing our paper. We have taken your suggestions to improve language into consideration and have made the necessary changes.

**2. In the lines 83-88 you mention the length of the training in months. Whose training duration is mentioned? As you mention in the next sentence that trainers also have to complete a training course, it is not clear to me if the length of the training is related to the trainer or the trainee.**

Response: Thank you for your comment. We adapted on line 87:

" The length of the training for residents in months, calculated on the basis of full-time employment, averaged longer than 6 months. All GPs were required to attend a training course before assigned a resident."

**3. In line 123 you mention the use of a qualitative approach. Which qualitative approach did you use in analysing your data? (e.g. exploratory, thematic analysis, etc.)**

Response: We are glad to clarfiy: We were open to see reasons of the participants for choosing primary care and therefore included open-ended questions that we analyed in an exploratory analysis. We added this information in line 127.

**4. For consideration: would you advise to implement GP-internships for medical students in order to raise the attention for general practice already during the course of the medicine study?**

Response: We share the hypothesis with the Reviewer. In Switzerland, we see almost all universities having implemented GP internships but no firm evaluation to estimate the effect on career choices.

**Reviewer 2:**

**1. This article provides an interesting review on a new GP training programme. Please see suggestions below:**
 **Abstract line 3
May be good to explain the term “canton” when first mentioned.**

Response: Thank you for your comment. On page 4 in line 39 canton is explained as being a geopolitical area equivalent to a state. We did not explain canton in the abstract, because of word count restrictions and kept the abstract concise and factual.

**2. Background line 53
You refer to general medicine as a specialty, rather than general practice/family medicine. In the UK general medicine is a separate specialty from general practice and it would be good to clarify if the issues you are referring to apply to all generalist fields e.g. general medicine in hospital and general practice.**

Response: In Switzerland general internal medicine is a medical speciality. After having obtained board certification for this specialty, medical doctors are allowed to work in various fields of general medicine, such as in a hospital setting or a general practice. General practice though is not a separate specialty.

To be more clear, we adapted on page 5 in lines 52-56: “

Additionally, there is a negative perception of general practice in the medical field, making it one of the least popular medical specialties. This is perceived in various Western countries and influences the decision-making of residents so that a system produces doctors who disproportionally avoid general practice [6].

**3. Methods line 85
Need to insert comma after training: “For training residents were assigned to a general practitioner who provided clinical teaching and supervision in a GP practice.”**

Response: adapted.
 **4. Methods line 88
Add in “being” between “before” and “assigned”.
“GPs were required to attend a training course before assigned a resident.”**

Response: adapted.

**5. Survey line 99
Insert apostrophe in “mentor’s”.
“importance of their GP training as well as their mentors influence in becoming a GP.”**

Response: adapted.

**6. Survey line 103
Insert full stop after “GPs.”:
“factors that influenced the career choice of GPs. Two authors categorized answers by the”**

Response: adapted.

**7. Baseline characteristics line 137
Change comma to full stop: “(38,8%)”**
Response: adapted.

**8. Career choices line 144
“15 (10 %) of trainees did not perceive a career in General Practice.” Change “did not perceive” to “did not expect to enter” or “did not want to enter a career in general practice”.**

Response: adapted.

**9. Reasons to become GPs line 164
“were following topics”. Insert “were THE following topics”.**

Response: adapted.


**10. Location of new GP practices line 178
“The proportion of the communities correlated on a whole to the increase of GPs starting their practices in that given area.”
I think this should say” correlated on a whole to the number of GPs” rather than “increase of GPs”.**

Response: adapted.

**11. Do you have figures for how many GPs per population there were before the study to show that the proportion of GPs working in rural areas was previously lower?**
Response: We agree with the Reviewer that this information would be really helpful. We find it challenging to explain to colleagues from different countries, that Switzerland has a huge problem in providing those data. E.g. in the Canton of Bern there is no up-to-date registry on active GPs which makes it impossible to have an estimate on previous numbers of GPs by geographical region.

**12. Discussion line 187
“Firstly, these results indicate that a vocational training program in a general practice increases the likelihood of a career in general practice.”
To say that it increases the likelihood you would need baseline figures, given there was not a previous training programme this is difficult to determine but perhaps looking at the number of new GPs starting in the area pre and post the training scheme?**

Response: We agree with the concern of the reviewer and given our lack of data as described above, we rephrased on line 203-205:

" Firstly, these results indicate that up to 10 years after a vocational training program in a general practice a large majority of 81% of the trainees had become GPs or were on track [10]. "

**13. Discussion line 197
“Group practices lead to reduced work-load, better resource-sharing and a better cooperation with GPs in a practice.”
Please add references to these points.**

Response: We added two new references (our reference 12 and 13).

**14. Implication for research and practice line 234
“mentoring can be more effective so that training residents becomes standardized”
Should this be “residencies” or “traineeships” rather than “residents”?**

Response: adapted.
