# Supplementary material for: Evaluating 10 years of state-funded GP training in GP offices in Switzerland
Source: PLoS One. 2020 Aug 17;15(8):e0237533. doi: 10.1371/journal.pone.0237533 (PMC7430752; doi:10.1371/journal.pone.0237533)
Supplement: Attachment — Submitted filename: Responsetoreviewers.docx [file pone.0237533.s004.docx]

Professor Sven Streit, MD, MSc, PhD

Professor in Primary Care

Head of Interprofessional Primary Care
Institute of Primary Health Care Bern (BIHAM)

University of Bern
Mittelstrasse 43
3012 Bern

Switzerland
Tel +41 31 631 58 75
Email: sven.streit@biham.unibe.ch

**Revision of manuscript ID:** PONE-D-20-09608R1

Dear Dr Soundy, dear PLOS ONE Editorial Board,

We were invited to submit a 2nd revision version of our manuscript:

**“Evaluating 10 years of state-funded GP training in GP offices in Switzerland**

We are glad to see that our revision satisfied the two reviewers. However, we appreciate the opportunity to address all their remaining comments.

Sincerely,

Sven Streit

**Reviewer 1**

**1. Thank you for your revision of the manuscript. You have reacted on all my concerns about the manuscript. I have one question left: Your data are available upon reasonable request, can you please indicate why your data are not fully available?**

Response: We thank the reviewer for the positive response. The cited statement on data availability was part of our first submission. However, when we submitted the revised manuscript, we were asked to by the Editorial team to make our data publically available which we did. We would like to point to page 14 line 266 where it says:

" All relevant data are within the manuscript and its Supporting Information files."

The file supporting information 2 contains all data.

**Reviewer 2:**

**1. Please see a few remaining minor comments: Abstract Page 2 line 23 ‘importance of their mentors influence’- insert apostrophe after mentors’**

Response: adapted

**2. Also for Page 10 line 165. Background Page 4 line 52 and 55- in response to reviewer comments you said you were changing reference to general medicine to ‘general practice’ but it still says general medicine.**

Response: our appologies, we adapted and rephrased "general practice" in line 52 and 55.

**Page 11 line 205 ‘Besides the possibility of part-time work (49.7%) our study shows most residents were female (67.9%) and trained in a dual or group practice (94.6%).’ Do you mean almost half of respondents were attracted to general practice by the possibility of part-time work?**

Response: As explained in line 213-214 we know from a previous study (our reference 14) that working part-time is a factor that young doctors are attracted to general practice.

**Page 12 line 217- ‘speaking one or both of the four major languages (German and French)’- should this say ‘one or two of’.**

Response: we adapted in line 219 "one or two of the four major languages".

**Page 12 line 222- ‘und’ should be ‘and’.**

Response: well spotted. We again searched the complete documents for typos and adapted in line 224 "and" instead of "und".
